# Increasing Dietary Potassium Chloride Promotes Urine Dilution and Decreases Calcium Oxalate Relative Supersaturation in Healthy Dogs and Cats

**DOI:** 10.3390/ani11061809

**Published:** 2021-06-17

**Authors:** Esther Bijsmans, Yann Quéau, Vincent Biourge

**Affiliations:** Royal Canin Research & Development Center, 34070 Aimargues, France; yann.queau@royalcanin.com (Y.Q.); vincent.biourge@royalcanin.com (V.B.)

**Keywords:** urolithiasis, urine crystallization, salt

## Abstract

**Simple Summary:**

Bladder- and kidney stones are common in dogs and cats, and urine dilution is a strategy often used to decrease the risk of stone and crystal-formation. Pet foods containing sodium chloride (NaCl, table salt) increase drinking with the resultant dilution of urine. The salt substitute potassium chloride (KCl) is a potential alternative for patients that would benefit from dietary sodium restriction, but the effects of KCl on urinary parameters is unknown. This study fed two dry pet foods differing only in KCl to healthy dogs and cats. When fed the diet containing KCl, dogs and cats increased their water intake, and urine volume increased. The urine was more dilute, and measures of calcium oxalate stone risk decreased. A pet food containing KCl is therefore an interesting alternative to NaCl and a novel nutritional strategy for the prevention of urinary stones.

**Abstract:**

Urine dilution is a strategy used to decrease the risk of crystallization in cats and dogs at risk of urolithiasis. Sodium chloride has been used in prescription diets to effectively promote urine dilution, but the effect of the salt-substitute potassium chloride (KCl) on urine parameters has not been extensively investigated. Two diets differing only in KCl (Diet A; K 0.44 g/MJ, Diet B; K 1.03 g/MJ) were fed to 17 cats and 22 dogs for seven days, followed by three days of urine collection. Urinary ion concentrations were determined by ionic chromatography, and SUPERSAT software was used to calculate the relative supersaturation (RSS) value for struvite and calcium oxalate. Water intake and urine volume increased, and USG decreased on diet B (*p* < 0.001). Urine concentration of potassium increased on diet B, but concentrations of all other ions did not change or decrease in line with urine dilution. Calcium oxalate RSS decreased on diet B (*p* < 0.05). This short-term study showed that increased dietary KCl in a dry extruded diet effectively dilutes the urine of cats and dogs and therefore offers a novel nutritional strategy for the prevention of urolithiasis. This finding is of interest for patients that would benefit from dietary sodium restriction.

## 1. Introduction

The two most common types of urinary stones in dogs and cats are struvite (magnesium ammonium phosphate) and calcium oxalate [1]. Urolithiasis accounts for 7–22% of cases of lower urinary tract disease in cats [2,3,4], and 18% of cases of lower urinary tract disease in dogs [5].

Nutritional strategies in animals aim to decrease the degree of supersaturation of urine with precursors of crystals and uroliths. The best estimation of urinary crystal formation is by assessing the first step of lithogenesis and measuring the relative supersaturation (RSS) value, which takes into account the urine concentration of various ions and urine pH [6]. A decrease in RSS can be achieved by limiting the amount of stone precursors excreted in the urine, or their concentration through urine dilution.

The most common dietary strategies in pets to promote urine dilution focus on increased moisture or sodium chloride (NaCl) to drive water intake [7]. Potassium chloride (KCl) is a salt substitute often used for humans that need to decrease their sodium intake [8]. A study performed in cats reported a gradual, but not statistically significant, increase in urine volume when increasing added K from 0.5% to 1% in the form of KCl [9]. Studies in humans [10], rats [11], and poultry [12] have shown that increased dietary KCl increases water intake. An increase in urinary excretion of both sodium and potassium is seen in rats in response to dietary potassium, and this is associated with a marked diuresis [13]. Additionally, increasing potassium intake decreases hypercalciuria in humans [14]. The combination of these factors makes KCl an interesting alternative to NaCl in commercially available diets formulated to manage urolithiasis risk, especially for animals that would benefit from lower sodium intakes.

This study aimed to investigate the effect of two dietary concentrations of KCl on water intake, urine volume, urine specific gravity (USG), the excretion and concentration of different urinary minerals and struvite and CaOx RSS in healthy dogs and cats. The hypothesis was that increased KCl intake would promote urine dilution and decrease RSS.

## 2. Materials and Methods

### 2.1. Animals

Seventeen healthy adult cats and 22 healthy adult dogs were included in the study. Daily observations by animal keepers and yearly veterinary check-ups (including physical examination, complete blood count and serum biochemistry panels, urinalysis, and diagnostic imaging) ascertained the health status of the animals. The cat panels included 11 male and six female cats; all were neutered. All dogs were female. The cat breeds represented included Domestic Shorthair (*n* = 7), British Shorthair (*n* = 5), Maine Coon (*n* = 2), American Shorthair (*n* = 1), Selkirk Rex (*n* = 1), and Highland Fold (*n* = 1). Ten dogs were Toy Poodles, seven were Miniature Schnauzers, and five were Dachshunds (one long-haired and four short-haired). At the beginning of the study, the cats were 3.7 ± 0.9 years old and weighed 5.2 ± 1.1 kg. The dogs were 5.4 ± 2.0 years old and weighed 5.5 ± 1.7 kg. The cats and dogs were maintained in a temperature-controlled (22 °C) facility with natural daylight. During the adaptation phase to the diet, the cats were housed collectively, but were individually housed during the 72 h of urine collection in lodges to which they had been acclimated prior via a habituation program. The dogs were individually housed in lodges during both the adaptation and urine collection phase. Ethical approval for the protocol was obtained from the internal Royal Canin Ethics Committee, and the housing and urine collection method were in agreement with the Mars Welfare Standards for Cat and Dog Facilities and received approval of the French Government (reference 1595.01).

### 2.2. Study Design and Procedures

#### 2.2.1. Diets

Two experimental dry extruded diets were used for this study. Both diets were assigned code-names, and the animal technicians and laboratory technicians were blinded as to what the purpose of each diet was. The study was designed as a cross-over study, and to exclude an effect of diet-order, eight dogs and eight cats were fed diet A first, followed by diet B, whereas nine cats and 14 dogs were fed diet B first, followed by diet A. One single batch of each diet was used for the entire trial. Diets were formulated to be nutritionally complete and balanced for adult dogs and cats, according to NRC recommended allowances [15], AAFCO (Association of American Feed Control Officials), and FEDIAF (European Pet Food Industry Federation) nutrient profiles. The two diets produced were identical, except for the addition of 1.9% KCl to diet B (base concentration KCl 0.6%) in exchange of corn flour, to achieve predicted levels of potassium of 0.41 g/MJ (diet A) and 1.12 g/MJ (diet B) (1.7 g/1000 kcal and 4.7 g/1000 kcal, respectively).

Ingredient and nutrient profiles of the two diets are reported in Table 1. The diets were analyzed for ash and DM by drying to a constant weight at 103 °C followed by combustion at 550 °C. Crude protein (adapted from NFENISO 16634-1) [16], crude fat (European regulation (CE) No. 152/2009 by extraction and filtration) [17], total dietary fiber (AOAC 985.29 by enzymatic reaction) [18], Ca, Na, Mg, K, and P (inductively coupled mass spectrometry), and Cl (European regulation (CE) No. 152/2009 by titration) [17] were determined. Nutrient analyses of the diets met the predicted levels.

#### 2.2.2. RSS Testing

All animals were fed each diet for seven days of adaptation, followed by three days of urine collection. Cats were fed individually using an electronically controlled door-system operated by a chip (Metal Process SAS, Montevrain, France) during the adaptation phase. Food was refreshed once daily, and quantities fed were based on the individual’s energy requirements in order to maintain body weight. All food offered and refused was weighed and recorded daily during the urine collection phase. Drinking water was offered ad libitum and intake was measured by recording how much was offered and how much was left when the water was refreshed. The urine collection phase took place in individual lodges. Animals were habituated to urinate on a plastic tray, without litter, whose sides were slightly included toward a collecting hole. All urine was collected via natural voiding into a clean Erlenmeyer flask. Animal caretakers checked these flasks multiple times per day and checked visually for contamination with feces. Absence of bacterial contamination was ascertained via duplicate pH measurements at 2-h time intervals and urine was only pooled if visually clean and pH remained within 0.25 units. The 3-day pooled urine sample was collected in a bottle containing 1 mL of 20% chlorhexidine (Hibitane; Mölnlycke Health Care, Gothenburg, Sweden) and was stored at 4 °C.

#### 2.2.3. Urine Analysis

A record of the weight, urine specific gravity (refractometer 30PX, Mettler Toledo AG, Greifensee, Switzerland), and pH (calibrated pH meter SEVENcompact, Mettler Toledo AG, Switzerland) of each urine sample and the final pool was kept for each animal on each diet. Urine volume was calculated by dividing the weight by the urine specific gravity.

Ion concentrations were determined on an aliquot from the urine pool, which was titrated to pH 2.0 with 37% hydrochloric acid in order to dissolve all salts. Samples were analyzed immediately. The concentrations of calcium, phosphate, magnesium, sodium, potassium, ammonium, oxalate, citrate, sulfate, and uric acid in the urine pool were measured by ionic chromatography (Dionex, Port Melbourne, Australia) as described in [19]. This method was determined to have a variability of less than 5% for the mineral ions and uric acid, and of 10% for the organic ions (oxalate and citrate). The computer software Supersat was used to calculate the magnesium ammonium phosphate (MAP) and RSS CaOx from the pH and urine concentrations of the ions above-mentioned [6]. Urinary excretion of all minerals was calculated as follows: (urine mineral concentration) × (3-day urine volume/3)/(body weight).

#### 2.2.4. Statistical Analyses

G*Power freeware [20] was used for the power calculations. A minimum difference of 1 for CaOx RSS was deemed relevant, and a standard deviation of 0.990 for CaOx RSS was used, based on historic research data from the Royal Canin Kennel and Cattery. This calculated an effect size of 1.01. Two-sided significance using *p* < 0.05 and 95% power was assumed. This determined a group size of a minimum of 15 animals. Cats in this study were housed in panels of nine animals during the adaptation phase. Two panels were therefore included in the study. One cat could not complete the study because of the development of a corneal ulcer, which led to the inclusion of 17 animals in total. Dogs were housed in panels of eight animals. Three panels of dogs were used to account for potential exclusions due to contaminated urine samples or soft feces in response to the dietary change. This was done to ensure that the results would be available for the minimum number of dogs needed as determined by the power calculation. One dog could not complete the study because of lameness, and one dog did not eat the low potassium diet. Therefore, a total of 22 dogs completed the study.

JMP software (version 15.0.0, SAS institute Inc., Cary, NC, USA) was used for the statistical analyses. Normality of variables was assessed by plotting the data and their residuals and a Shapiro–Wilks test, and data were log-transformed if needed to ensure normality criteria were met. Data analysis was performed using a linear mixed model including diet, panel, and the diet–panel interaction as fixed effects as well as a random animal effect. In case of a significant panel–diet interaction, post-hoc comparisons were performed with Tukey’s HSD. If no significant panel–diet interaction was found, this was removed from the model as it confirmed the absence of a potential effect of diet-order. As the aim of this study was not to compare panels but diets, the model was run as a paired t-test to compare between diets in that case.

Data are expressed as least square means ± standard error (LSM ± SE) when residuals of the model were normally distributed, or median [interquartile range] when it was not the case. Significance level was set at *p* < 0.05.

## 3. Results

An overview of the data is presented in Table 2 and Table 3.

All animals remained healthy for the duration of the study. There was no evidence for a difference in caloric intakes among diets in cats (*p* = 0.89) nor dogs (*p* = 0.71).

### 3.1. Cat Results

The majority of urine samples for cats were pooled and used for RSS measurement (85% on diet A, and 83% on diet B). Non-pooled samples were contaminated (e.g., with feces or food) and in some cases, the cat did not urinate a sufficient amount for the sample to be pooled after urine specific gravity and pH measurement.

There was a significant increase in water intake (diet A: 28.5 ± 1.6 mL/day/kg; diet B: 33.8 ± 1.6 mL/day/kg, *p* < 0.0001) and urine volume (diet A: 11.2 ± 0.9 mL/day/kg; diet B: 16.2 ± 0.9 mL/day/kg, *p* < 0.0001) on the high KCl diet in cats. USG was also significantly lower with KCl supplementation (1.069 ± 0.002 for diet A: 1.058 ± 0.002 for diet B, *p* < 0.0001).

Urinary excretions of magnesium, potassium, and citrate significantly increased on the high potassium diet (all *p* < 0.05). Urinary potassium concentrations increased with dietary potassium (*p* < 0.0001). Urine concentrations of all other ions except citrate decreased significantly with increasing levels of dietary KCl.

There was no significant difference in struvite RSS between diets (*p* = 0.11). Calcium oxalate RSS was significantly lower on the high KCl diet (diet A: 3.24 ± 0.37; diet B: 2.59 ± 0.37, *p* < 0.05).

### 3.2. Dog Results

The majority of urine samples for dogs were pooled and used for RSS measurement (73% on both diets). The majority of the samples that were not pooled were overnight samples that the dogs defecated in.

Dogs had a significant increase in water intake (diet A: 43.9 ± 1.4 mL/day/kg; diet B: 50.3 ± 1.4 mL/day/kg, *p* < 0.001), and a significant difference between panels was found, with the Toy Poodle panel drinking significantly more than the Miniature Schnauzer panel (*p* < 0.001), with a significant diet–panel interaction (*p* < 0.05). Urine volume was significantly higher on the KCl supplemented diet (diet A: 19.7 ± 1.2 mL/kg/day; diet B: 27.3 ± 1.2 mL/kg/day, *p* < 0.001), and USG was significantly lower with KCl supplementation (1.049 ± 0.002 for diet A, 1.041 ± 0.002 for diet B, *p* < 0.001).

Urinary excretions of potassium, oxalate, and citrate increased on the high potassium diet. However, urinary concentrations of all ions apart from potassium (which increased) and citrate (which did not change) decreased (all *p* < 0.005).

A significant panel–diet interaction was found for water intake, calcium and citrate excretion, and citrate concentration. Miniature Poodles drank significantly more than the other two panels. On post-hoc comparison, the Miniature Poodle panel had higher calcium excretion on the low potassium diet than the other panels, and they responded differently to the high potassium diet than the other panels, with significantly higher citrate excretions and concentrations. Overall, no effect of diet was found on calcium, magnesium, sodium, ammonium, phosphate, sulfate, and uric acid excretion (all *p* > 0.05).

There was no significant difference in struvite RSS between diets (*p* = 0.23). Calcium oxalate RSS was significantly lower on the high KCl diet (diet A: 15.0 [9.5, 19.5]; diet B: 11.0 [9.1, 15.1] *p* < 0.05).

## 4. Discussion

This study shows that increasing dietary potassium from 0.41 g/MJ to 1.12 g/MJ via the inclusion of KCl in an extruded dry diet significantly increases urine volume and decreases USG in cats and dogs. The concentrations of most urinary ions decreased, in line with the effect on urine dilution. This resulted in a significantly lower RSS for CaOx. This might suggest that a pet food supplemented with KCl can be an alternative strategy to NaCl to dilute urine in cats and dogs, which could contribute to the prevention of urolithiasis, though long-term feeding studies would be needed to confirm.

Urine volume increased by 45% in cats and 43% in dogs, and was comparable to urine volumes reported on diets with 73% moisture (wet diets) in both cats [21] and dogs [22]. The diuretic effect of potassium was first described in 1679, and has since been confirmed in other studies [23]. One study in cats reported a significant increase in urine volume after feeding diets supplemented with KCl, though not in a dose-dependent manner. However, the diet with 1% K from KCl induced a urine volume that was 27% greater than the control diet [9]. Studies performed in the 1930s suggested that KCl is not directly dipsogenic, but promotes drinking by diuresis [10]. In the current study, both species showed a significant increase in water intake. Potassium has been reported to function as a natriuretic in addition to being a diuretic in other species including dogs [13,21]. An increase in urinary sodium excretion was not observed in the current experiment. With an average intake of ~95 kcal/kg BW^0.711^ for cats and ~105 kcal/kg BW^0.75^ for dogs, K intake was 2130 mg/day in cats and 1750 mg/day in dogs, which was lower than the supplementation given in the reported study (200 mEq of potassium, equivalent to 7800 mg/day) [24]. However, bodyweight of the dogs included in the latter study was not known, making interpretation challenging. A study in which cats were fed diets supplemented with KCl or KHCO_3_ did not report a linear increase in urinary sodium concentration [9]. It is therefore possible that there is a dose-dependent effect on sodium excretion that we did not observe in the current study.

One small meta-analysis showed that dietary potassium intake in humans is associated with a lower RSS for CaOx and a 33–56% decrease in CaOx stone risk [25]. However, the authors questioned whether the response was due to potassium alone or in combination with other factors such as an increased fruit and vegetable intake, potentially increasing water and citrate intake. The diets used in the current study differed solely in KCl, making other effects less likely. As expected, urinary potassium excretion and concentration increased on the high potassium diet.

Although the lower CaOx RSS associated with the high KCl diet was likely the consequence of decreased urinary concentrations of calcium and oxalate, the effect of other ions cannot be discounted. Both urinary magnesium and citrate excretions (but not concentrations) were increased in cats, and citrate excretion (but not concentration) was increased in dogs. Magnesium has the potential to complex with oxalate [26], and citrate with calcium. In humans, urinary potassium level is an independent predictor of urinary citrate concentration, but potassium supplementation is not associated with increased citrate excretion [27,28,29]. Urinary oxalate excretion (but not concentration) increased in dogs on the high KCl diet. Oxalate can complex metallic ions including K+ and thus theoretically affect CaOx RSS [30]. Both species had higher urinary potassium concentrations, which is likely a direct reflection of the increase in dietary potassium. The reasons for the increased urinary excretions of the electrolytes described above in the animals in the current study are unclear. However, urinary magnesium, citrate, and oxalate concentrations decreased due to urine dilution, and any potential effect from this increased excretion was likely minimal.

Struvite RSS did not significantly differ between diets but was in the undersaturated zone for both [6]. Both diets had the same nutritional composition apart from KCl, and no difference in base excess. A urine pH <6.2 has been reported to promote a struvite RSS < 1 [7], and with a urine pH of ~6 for both diets in both species, the low struvite RSS values are in line with the expectations. Dietary base excess and urinary pH are more important predictors of struvite crystallization than dietary mineral intake such as magnesium [31,32]. The clinically similar urine pH between the two diets might therefore explain why struvite RSS did not differ, despite the lower concentrations of precursors on the high KCl diet.

In dogs, some differences between panels and their response to diet was found. The different response came consistently from the Miniature Poodle panel, which drank more than the Miniature Schnauzer and the panel that consisted of a mix of breeds and had higher urinary calcium excretions on the low potassium diet than a mix of the breeds. They also had higher urinary citrate excretion in response to the high potassium diet than the other two panels, though not to the extent that it affected CaOx RSS. Miniature poodles have been described to be predisposed to CaOx urolithiasis [33], but Miniature Schnauzers (one of the other panels included in the current study) is the breed that CaOx uroliths are most commonly reported in [33,34,35,36]. The finding that the miniature poodles had higher baseline urinary calcium excretion than other breeds is of interest, considering that higher urinary calcium excretion is a risk factor for calcium oxalate urolithiasis in dogs [37,38].

There are some limitations to this study. Only two levels of dietary KCl were compared, and diets were fed for 10 days each, which is a relatively short time period. Additionally, only urine samples were collected, and blood samples could provide extra insights into the response to the diet supplemented with KCl. It cannot be excluded that there are longer-term metabolic adaptations that will take place in addition to the rapid changes that were seen in urinary variables. However, there is evidence in rats that excretion of potassium plateaus after seven days of oral potassium loading [13]. The study was performed in healthy animals, and long-term studies in stone-forming animals are needed to assess the relationship between RSS and recurrence of urolithiasis. Urine was collected non-invasively throughout the study. Bladder catheterization would have given a more accurate measurement of urine volume, but this was not done for animal welfare considerations. The volume collected might therefore have been under- or overestimated (depending on the volume of urine in the bladder at the start and end of the study), impacting urinary excretion (but not concentration) calculations. All measures took place over the course of three days, and results were averaged to represent the values per 24 h. A longer collection period might have provided a more accurate average.

## 5. Conclusions

To conclude, this short-term study showed that increasing dietary KCl content in a dry extruded diet was effective in diluting the urine of cats and dogs. This was accompanied by a decrease in urinary mineral concentrations, and a decrease in CaOx RSS. A longer prospective study in stone-forming animals should be carried out to determine whether dietary KCl supplementation can prevent or delay recurrence of stone formation. Moreover, this dietary strategy should be used with caution in animals at risk of hyperkalemia such as those receiving potassium sparing medications.

## Figures and Tables

**Table 1:** Ingredient composition and analyzed nutrient profiles of the experimental diets.

Analyzed Nutrient	Diet A KCl 0.6%	Diet B KCl 2.5%
Moisture	7.97	7.70
Crude protein	19.6	20.1
Crude fat	6.9	7.1
Total dietary fiber	6.3	5.8
Ash	3.2	4.2
Calcium	0.42	0.40
Phosphorus	0.39	0.40
Magnesium	0.05	0.05
Potassium	0.44	1.03
Sodium	0.24	0.23
Chloride	0.54	0.83
Metabolizable energy ^a^	15.4	15.4

All nutrient contents are expressed in grams per MJ, except for moisture, which is expressed in %, and metabolizable energy, expressed in MJ/kg as fed. Ingredient composition by order of weight: corn and corn gluten, wheat and wheat gluten, dehydrated poultry protein, hydrolyzed animal proteins, rice, animal fats, corn flour, chicory pulp, minerals, egg powder, fish oil, soya oil, psyllium, fructo-oligosaccharides, glucosamine; ^a^ calculated with the cat predictive equations from NRC 2006 using total dietary fiber.

**Table 2:** Diet and water intakes, and urine variables in cats (*n* = 17) fed the two diets differing in potassium chloride content.

Variable	Diet A KCl 0.6%	Diet B KCl 2.5%	SE	*p*-Value for Diet	*p*-Value for Panel	*p*-Value for Panel × Diet
Caloric intake (kcal/BW^0.711^)	94.9	94.4	4.7	0.892	-	-
Water intake (mL/kg/24 h)	28.5	33.8	1.6	<0.0001	-	-
Urine volume (mL/kg/24 h)	11.2	16.2	0.9	<0.0001	-	-
Urine specific gravity	1.069	1.058	0.002	<0.0001	-	-
Urine pH	5.93	5.93	0.06	0.877	-	-
CaOx RSS	3.24	2.59	0.37	0.034	-	-
MAP RSS	0.29	0.17	-	0.112	-	-
Urinary concentrations (mmol/L)	-					
Calcium	0.9	0.7	0.1	0.046	-	-
Magnesium	5.6	5.2	0.4	<0.0001	-	-
Sodium	175.5	127.3	6.5	<0.0001	-	-
Potassium	203.3	350.5	11.8	<0.0001	-	-
Ammonium	298.99	217.77	10.57	<0.0001	0.997	0.036
Phosphate	78.1	58.0	3.1	<0.0001	-	-
Sulfate	164.7	112.3	5.2	<0.0001	-	-
Oxalate	1.83	1.46	0.10	<0.0001	0.58	0.037
Citrate	2.5	2.3	0.4	0.40	-	-
Uric acid	0.8	0.6	0.1	<0.0001	-	-
Urinary excretions (µmol/kg/24 h)	-					
Calcium	9.8	9.5	-	0.075	-	-
Magnesium	62.2	82.5	6.0	0.0002	-	-
Sodium	1942	2027	130	0.548	-	-
Potassium	2181	5895	-	<0.0001	-	-
Ammonium	3174	3428	-	0.294	-	-
Phosphate	852	910	46	0.205	-	-
Sulfate	1804	1769	94	0.714	-	-
Oxalate	20.1	21.6	1.1	0.323	-	-
Citrate	21.7	29.6	-	0.024	-	-
Uric acid	9.0	7.7	-	0.605	-	-

Data are presented as least square means except for caloric intake, MAP RSS, and calcium, potassium, ammonium, citrate, and uric acid excretions, for which medians are indicated. All variables presented as /24 h were measured over 72 h and divided by 3 to give a daily average for presentation in this table. Missing *p*-values represent an absence of significance in the mixed-effects model and these effects were therefore removed. BW; body weight, SE; standard error, kg; kilogram, h; hours.

**Table 3:** Diet and water intakes, and urine variables in dogs (*n* = 22) fed the two diets differing in potassium chloride content.

Variable	Diet A KCl 0.6%	Diet B KCl 2.5%	SE	*p*-Value for Diet	*p*-Value for Panel	*p*-Value for Panel × Diet
Caloric intake (kcal/BW^0.75^)	108.1	106.9	3.4	0.71	-	-
Water intake (mL/kg/24 h)	43.9	50.3	1.4	<0.001	<0.001	<0.05
Urine volume (mL/kg/24 h)	19.7	27.3	1.2	<0.001	-	-
Urine specific gravity	1.049	1.041	0.002	0.0002	-	-
Urine pH	5.92	6.06	0.07	0.025	-	-
CaOx RSS	15.0	11.0	n.a.	0.041	-	-
MAP RSS	0.20	0.15	n.a.	0.23	-	-
Urinary concentrations (mmol/L)	-					
Calcium	4.12	2.53	n.a.	0.0023	-	-
Magnesium	6.03	4.41	0.42	<0.0001	-	-
Sodium	136.3	94.0	6.1	<0.0001	-	-
Potassium	138.02	226.55	n.a.	<0.0001	-	-
Ammonium	215.4	159.1	9.2	<0.0001	-	-
Phosphate	49.6	37.0	3.0	0.0005	-	-
Sulfate	126.8	89.9	5.5	<0.0001	-	-
Oxalate	1.24	1.04	n.a.	<0.0001	-	-
Citrate	0.07	0.08	n.a.	0.09	0.08	<0.0001
Uric acid	1.08	0.81	n.a.	<0.0001	-	-
Urinary excretions (µmol/kg/24 h)	-					
Calcium	71.35	69.15	n.a.	0.60	0.03	0.02
Magnesium	109.7	114.7	7.5	0.34	-	-
Sodium	2557.4	2513.6	130.1	0.79	-	-
Potassium	2515.82	5793.43	n.a.	<0.0001	-	-
Ammonium	4072.3	4172.6	182.2	0.67	-	-
Phosphate	920.3	951.9	52.8	0.70	-	-
Sulfate	2378.5	2354.6	91.4	0.84	-	-
Oxalate	25.42	30.15	n.a.	0.0004	-	-
Citrate	1.39	2.04	n.a.	<0.0001	0.001	<0.0001
Uric acid	22.6	23.3	1.6	0.47	0.0005	-

Data are presented as least square means except for CaOx RSS, MAP RSS, and calcium, potassium, oxalate, citrate, and uric acid concentrations and calcium, potassium, oxalate, and citrate excretions, for which medians are indicated. All variables presented as /24 h were measured over 72 h and divided by 3 to give a daily average for presentation in this table. Missing *p*-values represent an absence of significance in the mixed-effects model and these effects were therefore removed. BW; body weight, SE; standard error, kg; kilogram, h; hours, n.a.; not applicable.

## Data Availability

The data presented in this study are available on request from the corresponding author.
